# Impact of Mindfulness Training on Spanish Police Officers’ Mental and Emotional Health: a Non-Randomized Pilot Study

**DOI:** 10.1007/s12671-022-01827-5

**Published:** 2022-01-14

**Authors:** Jaime Navarrete, Miguel Ángel García-Salvador, Ausiàs Cebolla, Rosa Baños

**Affiliations:** 1 Department of Personality, Evaluation, and Psychological Treatments, University of Valencia, Avd. Blasco Ibañez 21, 46010 Valencia, Spain; 2 Asociación H Policía, Madrid, Spain; 3 CIBER of Physiopathology of Obesity and Nutrition (CIBEROBN), Madrid, Spain

**Keywords:** Mindfulness-based intervention, Police officers, Psychopathology, Quality of life, Suicide ideation, Linear mixed models

## Abstract

**Objectives:**

The purpose of this exploratory non-randomized controlled study was to determine the acceptance and effectiveness of an 8-week mindfulness-based intervention (MBI) co-designed by a police officer.

**Methods:**

A pretest-posttest control group design was followed. Participants (MBI group = 20; control group = 18) answered baseline and post-training self-reported measures. In addition, the weekly emotional state of the MBI group was collected. Paired-samples *t*-test and analysis of covariance were performed for pre-post within-group and between-group differences, respectively, as well as linear mixed effects analysis of repeated measures for week-by-week data.

**Results:**

High acceptance and attendance rates, as well as significant pre-post within-group differences in the MBI group in mindfulness (*η*^2^ = 0.43), self-compassion (*η*^2^ = 0.43), depression (*η*^2^ = 0.54), anxiety (*η*^2^ = 0.46), stress (*η*^2^ = 0.51), difficulties in emotion regulation, sleep quality (*η*^2^ = 0.57), and burnout (*η*^2^ = 0.31–0.47), were identified. Moreover, police officers who underwent the MBI experienced a week by week decrease of anger, disgust, anxiety, sadness, and desire. Finally, after adjusting for pre-test scores, significant between-group differences were found in the way of attending to internal and external experiences (observing mindfulness facet; *η*_*p*_^2^ = 0.21), depression symptoms (*η*_*p*_^2^ = 0.23), general distress (*η*_*p*_^2^ = 0.24), and the degree of physical and psychological exhaustion (personal burnout; *η*_*p*_^2^ = 0.20).

**Conclusions:**

The preliminary effectiveness of this MBI on psychopathology and quality of life outcomes in Spanish police officers was discussed. Previous evidence regarding the promising use of MBIs in this population was supported.

Police are a social institution that constantly adapt to the problems of public safety and seek to offer the best strategies for reducing crime and disorder (Willis, 2014). In the performance of their duties, police officers are often exposed to violence, suffering, and death, which have profound consequences for their mental health (Violanti et al., 2017). In this line, Giessing et al. (2020) developed an ecological momentary assessment of stress during daily service over 3 weeks and described how police service might constitute a trigger of chronic work stress. Furthermore, critical incidents in policing, as those detailed in the aforementioned study, predict drinking behavior and posttraumatic stress disorder symptoms (Ménard & Arter, 2013). In addition, police officers experience poor sleep quality associated with night shifts and years of service (Garbarino et al., 2019). In addition, the exposure to chronic work stressors results in the psychological syndrome of burnout (Leiter et al., 2015), which has a high prevalence among police officers (Backteman-Erlanson et al., 2013; De la Fuente et al., 2013; Queirós et al., 2020; Schaible & Six, 2016).

Stressors of police work may come not only from operational aspects, such as traumatic events or work schedules, but also from organizational stressors, such as lack of reward or punishment for infractions, which could even be perceived as a greater source of stress (Shane, 2010). For instance, reduced decision making, low social support at work, and low rewards are risk factors of depression, anxiety, and burnout for police officers (Chan & Andersen, 2020; Sherwood et al., 2019). In fact, the prevalence of depression, anxiety, and post-traumatic stress disorder symptomatology seems an occupational concern among officers. Nevertheless, they refuse to seek mental health support (Jetelina et al., 2020).

Arble and Arnetz (2017) proposed a theoretical framework of coping strategies and resources that first responders, including police officers, might use to deal with the outcomes associated with their work. The model, which was later replicated with only police officers (Arble et al., 2018), suggested that after exposure to stress they seek resilience and well-being through both approach coping strategies (facing and regulating emotional experience) and/or avoidance coping strategies (avoiding emotional experience). On the one hand, it seems that a flexible application of both types of coping strategies is the more adaptive way to cope well with adverse events (Park et al., 2015). On the other hand, avoidant coping might result in worse mental health, given that it is associated with greater substance use and indirectly promotes worse well-being in police officers, who use avoidance more frequently than other non-military first responders (Arble et al., 2018). In this regard, experiential avoidance is described as a maladaptive response to stressors consistently linked with psychopathology at long term and emotion dysregulation (Aldao et al., 2010; Naragon-Gainey et al., 2017).

In the police context, emotion dysregulation has been related to suicide ideation. Officers who are not able to regulate their emotions after exposure to work stressors are prone to have suicidal ideation (Heffer & Willoughby, 2018; Law et al., 2015). Several factors explain the increased risk for suicide, some of them are inherent in policing, such as the access to firearms, erratic shift schedules, and a focus on helping others at the expense of their own needs. Others are the potential consequent psychological impairments that come from the service, such as PTSD and burnout (Stanley et al., 2016). Previous literature has shown that mindfulness was inversely related to suicide ideation and moderated the association between neuroticism and suicide ideation (Lamis & Dvorak, 2014; Tucker et al., 2014). Thus, the effect of MBIs on this variable is plausible, though more research is needed (e.g., Chesin et al., 2015).

Despite the substantial burden of mental health problems in police officers (Syed et al., 2020), there is a lack of rigorous studies regarding the efficacy of stress management interventions in this population (Patterson et al., 2012, 2014). However, different mental health interventions for stress prevention in police officers have been tested (e.g., LaMontagne et al., 2016; McCraty & Atkinson, 2012). Taking into account the determinant role of emotion regulation to cope with the exposure to stressful events (Arble et al., 2018), mindfulness training may be a promising intervention, given that it promotes adaptive emotion regulation by a process of approach-oriented attention deployment (Farb et al., 2014). In fact, Krick and Felfe (2020) have recently shown that mindfulness-based interventions (MBIs) may also be feasible and effective for agentic and male-oriented cultures, such as police officers. Furthermore, they encouraged practitioners to implement MBIs in these contexts.

Few studies have been conducted to address police officers’ burden from a mindful emotion regulation approach (e.g., Fitzhugh et al., 2019; Hoeve et al., 2021; Trombka et al., 2021). Among them, Christopher et al., (2016, 2018) designed and applied a MBI among police officers, finding that the program reduced sleep disturbance, burnout, self-reported aggression, work-related stress, and difficulties with emotion regulation. Furthermore, Grupe et al. (2021) applied a similar MBI and demonstrated improvements in work stress, sleep quality, mental health outcomes, and post-traumatic stress disorder symptoms. Krick and Felfe (2020) examined effectiveness of a MBI using a non-selective sample of police officers and showed a reduction of strain, health complaints, and negative affect as well as improvements in selfcare, mindfulness, and heart rate variability as a physiological marker of emotion regulation. Márquez et al. (2021), for their part, conducted a MBI in a group of national police officers from Spain and identified significant improvements in compassion satisfaction and perceived stress.

As mentioned, further studies about the efficacy of mental health interventions in police officers are needed. Specifically, mindfulness training might be feasible, accepted, and effective for several mental health outcomes, though research in that regard is limited yet. For instance, no previous studies have reported how an MBI affects frequency of suicide ideation in police officers. It is positively associated with emotion dysregulation (Law et al., 2015) and highly prevalent in police officers (Violanti & Steege, 2021). Although there is promising effectiveness of MBIs on suicide ideation (Chesin et al., 2016; Forkmann et al., 2016), it has not been researched in police officers. Moreover, little is known about how to overcome the reluctance of police officers to enrol in MBIs (Krick & Felfe, 2020). In addition, researchers could take advantage of assessing their participants every week. Weekly measurement allows more detailed, sensitive, and wide-ranging assessments. In turn, this could enrich researchers’ conclusions, which usually are drawn on the basis of global pre- and post-assessments (Shiffman et al., 2008).

Therefore, this pilot study aimed to demonstrate the acceptability, adherence, and efficacy of an 8-week MBI (*Mindfulness Aplicado al Bienestar Policial*/Mindfulness to Promote Police Well-being) designed and implemented by a police officer (along with a psychologist) in Spanish police officers. The following hypotheses were tested: (1) The MBI will be well accepted by participants in terms of session attendance (6 or more sessions), medium-high adherence to meditation practice (at least 21/42 days), and good general opinion; (2) the MBI will show efficacy in increasing mindfulness, self-compassion, and sleep quality, as well as in decreasing depression, anxiety, perceived stress, burnout, and emotion dysregulation with moderate to large effect sizes, being both pre-post within-group differences in the MBI group and post-test between-groups differences (after adjusting for pre-test scores) statistically significant; (3) the MBI will show efficacy in decreasing frequency of suicide ideation with moderate to large effect sizes, being both pre-post within-group differences in the MBI group and post-test between-groups differences (after adjusting for pre-test scores) statistically significant; and (4) police officers who undergo the MBI (only assessed within the MBI group) will experience, from the first session, a gradual decrease of “negative emotions,” understood as aversive and uncomfortable emotional states, i.e., anger, disgust, fear, desire, sadness, and anxiety, as well as an increase of “positive emotions,” operationalized as pleasant emotional states, i.e., happiness and relaxation.

## Method

### Participants

**Figure 1:**
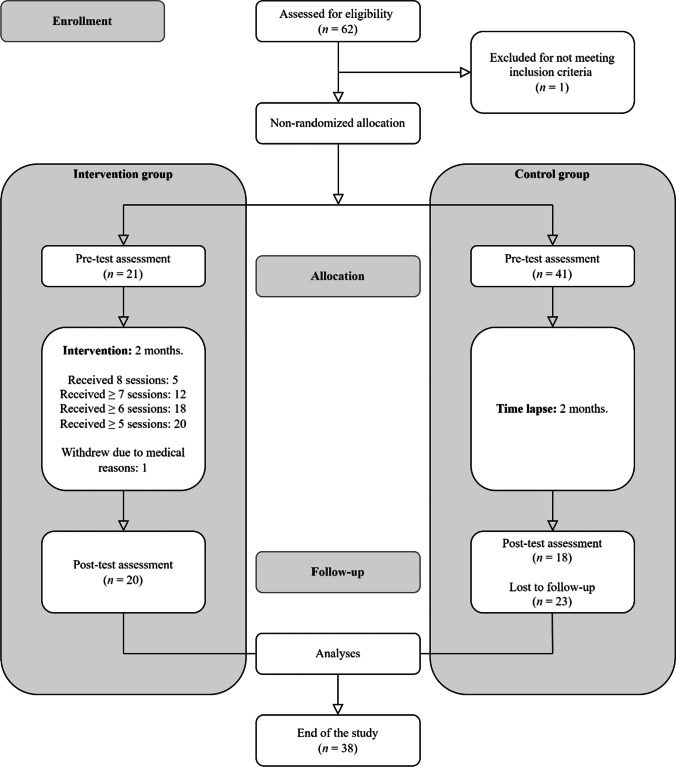
Flowchart of participants

The initial sample was composed of a total of 62 participants, police officers from the province of Valencia (Spain) who agreed to participate and were assigned to the intervention (MBI) group (*n* = 21) or the waiting list (WL) group (*n* = 41). To be eligible for participating in the study, they had to be already working as a police officer (any rank), and able to regularly attend the sessions. Double the number of participants in the WL group were enrolled because it was expected that many of them would withdraw. As shown in Table 1, the final sample was composed of 20 participants (*M*_age_ = 39; SD = 6.23; range 30–51 years) for the MBI group and 18 participants (*M*_age_ = 41.06; SD = 6.6; range 30–52 years) for the WL group. Figure 1 shows a flow diagram of participants’ progression through the study.

**Table 1:** Sociodemographic characteristics of final sample participants

Baseline characteristic	MBI condition		WL condition		Full sample	
	*n*	%	*n*	%	*n*	%
**Gender**						
Female	8	40	7	39	15	39
Male	12	60	11	61	23	61
**Marital status**						
Single	9	45	5	28	14	37
Married	9	45	8	44	17	45
Divorced	1	5	5	28	6	16
Widowed	1	5	0	0	1	2
**Highest educational level**						
Middle school	0	0	1	6	1	2
High school/some college	9	45	4	22	13	34
University or post-graduate degree	11	55	13	72	24	63
**Previous experience **^**a**^						
Meditation practice	7	35	5	28	12	32
**Law enforcement agency**						
National Police Corps	16	80	14	78	30	79
Municipal Police	4	20	4	22	8	21

*N* = 38 (*n* = 20 for participants of MBI condition and *n* = 18 for participants of WL condition). Participants were on average 39.97 years old (*SD* = 6.41). Participants of MBI condition were on average 39 years old (*SD* = 6.23). Participants of WL condition were on average 41.06 years old (*SD* = 6.60).

^a^ Reflects the number and percentage of participants answering “yes” to this question.

### Procedure

The Ethics Committee of Research in Humans of the Ethics Commission in Experimental Research of the University of Valencia approved the procedure (FF6DVJ7DQ97Y2112). Beginning in September 2020, the training was offered through social networks and advertising posters in the municipal police stations of the province of Valencia (Spain) with the permission of the provincial office. Interested participants had to email the research team and give information about their rank and to which police station they belonged to. Then, they were asked to provide information on their mental health (presence or not of a psychiatric diagnosis) and current employment status. The MBI group consisted of the first 21 officers that inscribed to the study. Therefore, the rest of the participants were allocated to the waiting list. Inclusion criteria were being active police officer and availability for attending the sessions (National Police Corps or Municipal Police). Exclusion criteria were as follows: presence of psychiatric disorder or being on sick leave. National Police Corps operates under the authority of central government, meanwhile Municipal Police is organized at the municipal level. Both police forces are responsible for policing urban areas, though they have distinct and specialized roles. For instance, the National Police Corps performs criminal investigation and Municipal Police deals with traffic.

A non-randomized pilot trial design was followed. In-person pre- and post-training assessments were held at the beginning of the first and last session. For that purpose, participants of the MBI filled out a pen and paper package. In addition, an emotional state measure and two feedback questions about at-home formal meditation practice were included per session (only for the MBI group). Simultaneously, participants from the control group were surveyed via the LimeSurvey platform of the University of Valencia which guarantees participant confidentiality and anonymity. All of them were properly informed about the study and consented to participate complying with the ethical guidelines set out in the Declaration of Helsinki.

Eight 2-h sessions were implemented each Thursday between October 15 and December 3, 2020, on double shifts (morning from 10 a.m. to 1 p.m. and afternoon from 4:30 a.m. to 6:30 a.m.). Participants could attend the course at the time that best adapted to their working schedules, though the sessions were limited to 11 individuals. All the sessions were carried out in the Faculty of Psychology of the University of Valencia following key rules to deal with coronavirus: each individual’s temperature was measured upon arrival, participants sat 1.5–2 m apart from each other, individuals wore a face covering, windows were completely open, and furniture was disinfected before and after each shift.

The current intervention (*Mindfulness Aplicado al Bienestar Policial*/Mindfulness to Promote Police Well-being) was designed to enhance emotion regulation capability in the context of police officers’ work. It was based on the Hölzel et al. (2011) model of mindfulness mechanisms, which proposes attention regulation, body awareness, emotion regulation, and changes in the perspective of the self as components through which mindfulness meditation works. Cebolla et al. (2018) empirically tested the relationship among these components and showed that attentional control positively affects both body awareness and emotion regulation strategies (mainly reappraisal and non-reactivity), which in turn directly promote a detached perspective of the self. Thus, the 8-week curriculum was designed to train participants in the attentional, constructive, and deconstructive meditation practices that evoke those mechanisms (Dahl et al., 2015): awareness of breath meditation, awareness of senses meditation, body scan, evaluative body scan, emotion regulation from body meditation, awareness of thoughts, emotion regulation from thoughts meditation, and self-inquiry meditation.

The mindfulness training development followed the pedagogical model of Cebolla and Campos (2016) for teaching mindfulness. The context of the intervention was psycho-educational, and the sessions were designed and administered by a psychologist (J.N.) and a police officer (M.G-S.), both specializing in group-format MBI. The teachers were trained in a local psychology center that provides specialized training and skills updating of mindfulness instructors (Elephant Plena, Valencia, Spain). They had 3 years’ experience in leading group-format MBIs. The structure of the sessions was as follows: (1) guided meditation; (2) an inquiry process; (3) an analysis of the week’s difficulties; (4) a brief theoretical explanation; and (5) guided meditation of the next daily homework meditation practice. For detailed information of each session content, see Table 2.

**Table 2:** Outline of the MBI intervention: summary of sessions, components, and topics covered.

MODULE 1: Attention regulation	
Session 1	
1. Lecture (15 min): Scientific framework of the intervention.	Introducing mindfulness and explaining the rationale and origins of the intervention.
2. Presentation of participants (10 min).	Each participant presented him or herself (name, work, and expectations).
3. Lecture (20 min): What is mindfulness?	Defining mindfulness, mindfulness mechanisms, mindfulness practice (formal and informal).
4. Meditation practice (5 min): The raisin meditation.	This practice complemented the theoretical explanation of mindfulness.
5. Instructions for homework meditation (20 min).	How to meditate at home: Finding the best place, finding the best moment.
6. Meditation practice (20 min): Awareness of breath meditation.	In this practice, participants were invited to direct mindful attention to breathing sensations.
Homework (once per day):	
- Awareness of breath meditation.	
- Awareness of pleasant activity.	In this practice, participants were invited to savor activities that they considered as pleasant (e.g., a walk).
Session 2	
1. Meditation practice (20 min): Awareness of breath meditation.	
2. Analysis of the week’s difficulties (40 min).	Exploring the difficulties that participants experienced when meditated during the week.
3. Minilecture (20 min): A brief revision of What is mindfulness?	The exposed difficulties are taken into advantage for revise the content of the last session.
4. Meditation practice (20 min): Body scan	In this practice, participants were invited to direct mindful attention to body sensations.
Homework (once per day):	
- Body scan meditation.	
- Awareness to a neutral activity.	In this practice, participants were invited to savor activities that they considered as neutral (e.g., driving).
MODULE 2: Body awareness	
Session 3	
1. Meditation practice (20 min): Awareness of senses meditation.	In this practice, participants were invited to direct mindful attention to what they could perceive through the five senses.
2. Analysis of the week’s difficulties (40 min).	
3. Lecture (20 min): Mindfulness of physical body.	Definition of body awareness, how to increase it with meditation.
4. Meditation practice (20 min): Evaluative body scan.	In this practice, participants pay attention to body sensations and classify them into pleasant, neutral or unpleasant.
Homework (once per day):	
- Evaluative body scan meditation.	
- Awareness to an unpleasant activity.	In this practice, participants were invited to savor activities that they considered as unpleasant (e.g., taking out the trash).
Session 4	
1. Meditation practice (20 min): Evaluative body scan.	
2. Analysis of the week’s difficulties (40 min).	
3. Lecture (20 min): Mindfulness of motion.	
4. Meditation practice (20 min): Standing meditation, slow walking meditation.	In this practice, participants are invited to direct mindful attention to body sensations and breathing while standing/walking.
Homework (once per day):	
- Body scan meditation.	
- Awareness to wearing the uniform.	In this practice, participants are asked for direct mindful attention to body sensations and thoughts while wearing/taking off the uniform.
MODULE 3: Emotion regulation (I)	
Session 5	
1. Meditation practice (20 min): Evaluative body scan.	
2. Analysis of the week’s difficulties (40 min).	
3. Lecture (20 min): Mindfulness of emotion (I).	How to regulate emotions from the body: exposure, extinction, and reconsolidation.
4. Meditation practice (20 min): Emotion regulation from body meditation.	In this practice, participants are invited to remember an unpleasant memory, to pay attention to the physical sensations related to this memory, and to direct mindful attention to them.
Homework (once per day):	
- Emotion regulation from body meditation	
- Awareness to wearing the uniform.	
MODULE 4: Emotion regulation (II)	
Session 6	
1. Meditation practice (20 min): Emotion regulation from body meditation.	
2. Analysis of the week’s difficulties (40 min).	
3. Lecture (20 min): Mindfulness of emotion (II).	How to regulate emotions from the mind: reappraisal through meditation.
4. Meditation practice (20 min): Awareness of thoughts.	In this practice, participants are invited to pay mindful attention to their minds and just observe with curiosity and acceptance their thoughts.
Homework (once per day)	
- Awareness of thoughts.	
- Awareness to wearing the uniform.	
MODULE 5: Perspective on the self	
Session 7	
1. Meditation practice (20 min): Awareness of thoughts.	
2. Analysis of the week’s difficulties (40 min).	
3. Lecture (20 min): The unchanging self and meta-awareness.	Deconstructing the permanent and unchanging self.
4. Meditation practice (20 min): Self-inquiry meditation.	In this practice, participants are invited to direct mindful attention to their thoughts, to choose one of them, maybe one that is specially frequent, and to inquiry him/herself about its importance to the self.
Homework (once per day):	
- Free choice of meditation.	
- Awareness to wearing the uniform.	
Session 8	
1. Meditation practice (20 min): safe place meditation.	In this practice, participants are invited to imagine a place in which they feel secure and to savor that moment.
2. Analysis of the week’s difficulties (40 min).	
3. Farewell speech.	

After receiving participants, they were asked for answering the weekly assessments every session. All meditations that were performed within sessions were followed of an inquiry about the experience during practice. Participants received audios (20 min) for the at-home meditations.

### Measures

#### Demographics

Basic demographics were gathered (age, gender, marital status, highest educational level, previous experience in meditation practice, and law enforcement agency).

#### Feasibility and Acceptability

General opinion after the intervention was assessed with an ad hoc questionnaire of 8 items (e.g., “I have learnt strategies to improve emotion regulation”) on a Likert-type scale from 1 (completely disagree) to 5 (completely agree). Scores above 4 were considered high acceptance/usefulness. In addition, in each session, two feedback questions about at-home formal meditation practice (“During the last week, how many days have you meditated?” and “During the last week, how many minutes per day have you meditated?”) were included. Session attendance was registered each session.

### Preliminary Effectiveness Outcomes

#### Discrete Emotions Questionnaire (DEQ)

The DEQ (Harmon-Jones et al., 2016) is a 32-item self-report measure designed to assess state self-reported emotions. It consists of eight subscales (four items per each one) corresponding to the “basic” emotions of anger (e.g., “Rage”), disgust (e.g., “Grossed out”), fear (e.g., “Panic”), sadness (e.g., “Lonely”), happiness (e.g., “Satisfaction”), anxiety (e.g., “Dread”), desire (e.g., “Craving”), and relaxation (e.g., “Chilled out”). Participants had to rate the extent to which they experienced them during the last week on a 7-point Likert scale from 1 (not at all) to 7 (an extreme amount). They answered this questionnaire every session. Internal consistency (Cronbach’s *α* or mean inter-item correlation, see “Data Analyses” section for more information) of anger (*M*_Session 1_ = 0.28; *α*_Sessions 2-8_ = 0.69 – 0.85), disgust (*M*_Session 1_ = 0.21; *M*_Session 4_ = 0.24; *M*_Session 6_ = 0.31; *M*_Session 8_ = 0.2; *α*_Sessions 2,3,5,7_ = 0.70 – 0.84), fear (*α*_Session1_ = 0.83; *α*_Session5_ = 0.88; *M*_Session6_ = 0.10; *M*_Sessions 2-4, 7-8_ = 0.28 – 0.41), sadness (*α*_Sessions 1-6, 8_ =0.75 – 0.84; *M*_Session 7_ = 0.22), happiness (*α*_Sessions 1-8_ = 0.82 – 0.95), anxiety (*M*_Session 1-4_ = 0.22 – 0.27; *α*_Sessions 5-8_ = 0.70 – 0.77), desire (*M*_Sessions 1-6_ = 0.13 – 0.27; *α*_Sessions 7,8_ = 0.78 – 0.83), and relaxation (*α*_Sessions 1-8_ = 0.80 – 0.95) subscales was overall adequate.

#### Five Facets of Mindfulness Questionnaire-Short Form (FFMQ-SF)

Bohlmeijer et al. (2011) proposed a short version of the Five Facets of Mindfulness Questionnaire (Baer et al., 2006). The Spanish validation of the FFMQ-SF (Asensio-Martínez et al., 2019) was used. It comprises 23 items with a 5-point Likert scale ranging from 1 (never or very rarely true) to 5 (very often or always true) and five subscales: observing (4 items; e.g., “I pay attention to physical experiences, such as the wind in my hair or sun on my face”), describing (5 items; e.g., “I’m good at finding words to describe my feelings”), acting with awareness (5 items; e.g., “I rush through activities without being really attentive to them”), non-judging internal experience (5 items; e.g., “I tell myself I shouldn’t be feeling the way I’m feeling”), and non-reactivity to internal experience (4 items; e.g., “I watch my feelings without getting carried away by them”). Internal consistency of all FFMQ-SF subscales among participants (*α*_pre_ = 0.71 – 0.92; *α*_post_ = 0.71 – 0.91) was adequate. A total score can be calculated by adding all subscale scores, which demonstrated excellent internal consistency (*α*_pre_ = 0.89; *α*_post_ = 0.92).

#### Self-Compassion Scale Short-Form (SCS-SF)

The SCS-SF (Raes et al., 2011) comprises 12 items (e.g., “I try to be understanding and patient towards those aspects of my personality I don’t like”) scored on a Likert-type scale ranging from 1 (almost never) to 5 (almost always). The Spanish validation of the SCS-SF (Garcia-Campayo et al., 2014) was used. In general, the unidimensional structure of 12 items shows good psychometric properties; however, following recent recommendations about the interpretation and scoring of the scale, separate scores for the Positive (Self-Kindness, Common Humanity, and Mindfulness) and Negative (Self-Judgement, Isolation, and Over-identified) subscales (López et al., 2015; Muris & Otgaar, 2020) were also calculated. Both the Positive (*α*_pre_ = 0.82; *α*_post_ = 0.83) and Negative (*α*_pre_ = 0.84; *α*_post_ = 0.87) subscales showed adequate internal consistency, as well as the total score (*α*_pre_ = 0.85; *α*_post_ = 0.87).

#### Depression, Anxiety, and Stress Scale (DASS-21)

The DASS-21 (Henry & Crawford, 2005) is the shorter version of the DASS (Lovibond & Lovibond, 1995). It is a 21-item self-report measure scored on a 4-point Likert scale ranging from 0 (did not apply to me at all) to 3 (applied to me very much, or most of the time). It comprises three independent scales: Depression (7 items; e.g., “1 felt that life wasn't worthwhile”), Anxiety (7 items; e.g., “I felt I was close to panic”), and Stress (7 items; e.g., “I found it difficult to relax”). A total score can be calculated by computing the mean of all subscale scores. The Spanish validation was applied (Daza et al., 2002) showing in our sample adequate internal consistency for the Depression (*α*_pre_ = 0.93; *α*_post_ = 0.92), Anxiety (*α*_pre_ = 0.86; *α*_post_ = 0.92), and Stress (*α*_pre_ = 0.89; *α*_post_ = 0.90) factors, as well as for the General Distress factor (*α*_pre_ = 0.95; *α*_post_ = 0.96).

#### Frequency of Suicidal Ideation Inventory (FSII)

The FSII (Chang & Chang, 2016) is a short scale which consists of five items (e.g., “Over the past year, how often have you thought about committing suicide?”) scored on a Likert-type scale ranging from 1 (never) to 5 (almost every day). The Spanish validation (Sánchez-Álvarez et al., 2020) was applied and showed adequate internal consistency (*α*_pre_ = 0.95; *α*_post_ = 0.99).

#### Patient-Reported Outcomes Measurement Information System-Sleep Disturbance (PROMIS-SD)

The PROMIS-SD (Cella et al., 2007) is a short scale developed by the National Institutes of Health comprised of 8 items (e.g., “I had difficulty falling asleep”) scored on a Likert-type scale ranging from 0 (never) to 4 (always). In the present sample, the Spanish version has shown adequate internal consistency for the total score (*α*_pre_ = 0.89; *α*_post_ = 0.93).

#### Copenhagen Burnout Inventory (CBI)

The CBI (Kristensen et al., 2005) is a 19-item questionnaire scored on a 5-point Likert Scale from 1 (never) to 5 (always) designed to assess: personal burnout (6 items; e.g., “How often are you physically exhausted?”), work-related burnout (7 items; e.g., “Do you feel worn out at the end of the working day?”), and client-related burnout (6 items; e.g., “Do you find it hard to work with clients?”). The Spanish version was applied (Molinero Ruiz et al., 2013). The CBI demonstrated adequate internal consistency for the personal burnout (*α*_pre_ = 0.94; *α*_post_ = 0.95), work-related burnout (*α*_pre_ = 0.83; *α*_post_ = 0.79), and client-related burnout (*α*_pre_ = 0.85; *α*_post_ = 0.89) subscales.

#### Difficulties in Emotion Regulation Scale-Short Form (DERS-SF)

The DERS-SF (Kaufman et al., 2016) is the 18-item version of the DERS (Gratz & Roemer, 2004) consisting of six subscales measuring limited access to emotion regulation strategies (3 items; e.g., “When I’m upset, I believe there is nothing I can do to make myself feel better”), nonacceptance of emotional responses (3 items; e.g., “When I’m upset, I feel guilty for feeling that way”), impulse control difficulties (3 items; e.g., “When I’m upset, I have difficulty controlling my behavior”), difficulties engaging in goal-directed behavior (3 items; e.g., “When I’m upset, I have difficulty getting work done”), lack of emotional awareness (3 items; e.g., “When I’m upset, I acknowledge my emotions”), and lack of emotional clarity (3 items; e.g., “I have difficulty making sense out of my feelings”) with responses ranging from 1 (almost never) to 5 (almost always). A total score can be calculated by adding all items. We used the Spanish validation form (Wolz et al., 2015). Internal consistency of all DERS-SF subscales among participants (*α*_pre_ = 0.83 – 0.94; *α*_post_ = 0.81 – 0.93) was adequate, except for awareness subscale (*α*_pre_ = 0.51; *α*_post_ = 0.50). However, the mean inter-item correlation was adequate (*M*_pre_ = 0.26; *M*_post_ = 0.26). In the current sample, the DERS-SF total score demonstrated excellent internal consistency (*α*_pre_ = 0.92; *α*_post_ = 0.90).

### Data Analyses

All the analyses were performed using IBM SPSS Statistics for Windows, Version 26, and JASP, Version 0.14.1.0. The internal consistency for the instruments was established by calculating the Cronbach alpha indicator. Coefficients above 0.70 were considered adequate (DeVellis, 2016). However, Cronbach’s alpha (*α*) usually shows low values with short scales (< 10 items), so we calculated the mean inter-item correlation for the short scales that failed DeVellis (2016) criterion. In these cases, Briggs and Cheek’s (1986) optimal range (*M*) from 0.2 to 0.4 for inter-item correlation was followed.

Basic demographics, adherence values, and general opinion were descriptively analyzed. A paired-samples *t*-test was conducted to study the efficacy of each condition (MBI/time lapse) on police officers’ pre-/post-test scores on the FFMQ-SF (mindfulness), SCS-SF (self-compassion), DASS-21 (depression, anxiety, stress), FSII (frequency of suicide ideation), PROMIS-SD (sleep quality), CBI (burnout), and DERS-SF (difficulties in emotion regulation). The eta squared effect size was used, with cutoff values of 0.01, 0.06, and 0.14 for small, medium, and large effect sizes, respectively (Cohen, 1988).

A two-way between-groups analysis of Covariance (ANCOVA) was conducted to compare the effectiveness of the MBI with control group’s time lapse while checking if having previous experience in meditation practice was acting as a moderator variable in influencing the effectiveness of the intervention. The independent variables were the type of intervention (MBI, WL) and previous experience in meditation practice (yes or no). The dependent variables consisted of post-test scores on the FFMQ-SF, SCS-SF, DASS-21, FSII, PROMIS-SD, CBI, and DERS-SF. Participants’ scores on the pre-intervention administration of those questionnaires were used as the covariate in this analysis.

Alpha level was set at .01 for *t*-tests and ANCOVA in order to minimize type 1 error. Preliminary checks were conducted to ensure that there was no major violation of the assumptions of normality, linearity, homogeneity of variances, homogeneity of regression slopes, or reliable measurement of the covariates.

To test for differences in self-report state emotions (DEQ), a linear mixed effects analysis of repeated measures data was conducted using JASP version 0.14.1.0. Each model included fixed effects of week (W1 – 8) and a random effect of participants with a random intercept. Model terms were tested with the Satterthwaite method. Post hoc comparisons with Bonferroni adjustment to the alpha level were conducted for emotions that showed a significant effect of week.

## Results

Results suggest that the MBI was generally accepted as evidenced by session attendance, with 86% of participants attending at least 6/8 sessions (except for 2 participants who attended 5; see Figure 1). Moreover, participants reported engaging in formal at-home practice on average 27/42 days (*SD* = 10.88), with an average of 23 min per day (*SD* = 8.57). Thus, hypothesis 1 was supported.

In general, participants regarded the training as helpful for them to improve attentional control (*M* = 4.65; *SD* = 0.81), emotion regulation (*M* = 4.40; *SD* = 1.10), and work-related stress management (*M* = 4.35; *SD* = 0.88). Moreover, not only did they believe that the program could be useful for other police officers (*M* = 4.85; *SD* = 0.49), but also for the general population (*M* = 4.50; *SD* = 0.89). Finally, participants felt that the MBI had helped them cope with their daily service (*M* = 4.40; *SD* = 0.68). In fact, they would like to keep practicing meditation after the study (*M* = 4.80; *SD* = 0.52). Overall, the 85% of participants who underwent the MBI reported scores equal or higher to 4 in those items.

The paired-samples *t*-tests for the MBI group showed that there was a statistically significant increase in total scores of FFMQ-SF (*p* = .001) and SCS-SF (*p* = .001) from pre-test to post-test assessment. The eta squared statistic indicated large effect sizes for all mindfulness and self-compassion variables (eta squared statistic = 0.09 – 0.43). Meanwhile, the control group remained unchanged for FFMQ-SF and SCS-SF scores. Table 3 shows means, standard deviations, and paired samples *t*-test in mindfulness and self-compassion facets.

**Table 3:** Means, Standard Deviations, Paired Samples t-test, and Two-Way Analysis of Covariance Comparing Mindfulness and Self-Compassion

Measure	MBI condition		*t* (19)	*p*	*η*^2^	WL condition		*t* (17)	*p*	*η*^2^	ANCOVA		
	Pre-test	Post-test				Pre-test	Post-test				*F*_(1, 33)_	*p*	*η*_*p*_^2^
FFMQ-SF (Total Scale)	74.55 (15.40)	85 (12.1)	−3.81	.001	0.43	79.67 (17.97)	83.39 (17.95)	−2.29	.035	0.24	5.90	.021	0.15
Nonreactivity	13.15 (3.59)	14.25 (3.35)	−1.36	.191	0.09	12.5 (3.31)	13 (3.58)	−0.85	.408	0.04	1.31	.260	0.04
Observing	11.5 (4.2)	14.5 (3.02)	−3.79	.001	0.43	11.56 (5.02)	11.83 (4.79)	−0.49	.633	0.01	8.57	.006	0.21
Actaware	15.35 (5.49)	17.7 (4.26)	−1.94	.067	0.17	16.22 (6.89)	18.50 (5.59)	−3.37	.004	0.40	0.23	.636	0.01
Describing	18.3 (4)	19.3 (3.6)	−1.45	.165	0.10	20 (3.56)	19.83 (4.87)	0.21	.839	0.00	1.55	.221	0.05
Nonjudging	16.25 (4.78)	19.25 (4.14)	−3.03	.007	0.33	19.39 (5.28)	20.22 (4.11)	−1.44	.168	0.11	1.76	.193	0.05
SCS-SF (Total Scale)	3.18 (0.79)	3.75 (0.73)	−3.77	.001	0.43	3.52 (0.80)	3.60 (0.88)	−0.88	.393	0.04	5.77	.022	0.15
Positive Subscale	3.09 (0.96)	3.53 (0.85)	−2.76	.012	0.29	3.03 (0.90)	3.15 (0.99)	−1	.331	0.06	4.11	.051	0.11
Negative Subscale	2.74 (0.99)	2.03 (0.83)	2.93	.009	0.31	1.99 (0.87)	1.95 (1.05)	0.32	.752	0.01	1.73	.197	0.05

Standard deviations are presented in parenthesis. *FFMQ-SF*, Five Facets of Mindfulness Questionnaire – Short-Form; *Actaware*, Acting with awareness; *SCS-SF*, Self-Compassion Scale – Short-Form. Data about main effects for group are reported. The interaction between group and previous experience in meditation practice was not significant for all variables.

Similarly, participants from the MBI group showed a statistically significant decrease in depression (*p* < .001), anxiety (*p* = .001), and stress (*p* < .001) with large effect sizes (eta squared statistic = 0.46 – 0.51), while the control group remained overall unchanged for all these outcomes. See Table 4 for more details.

**Table 4:** Means, standard deviations, and paired samples *t*-test, and two-way analysis of covariance comparing psychopathology and frequency of suicidal ideation

Measure	MBI condition		*t* (19)	*p*	*η*^2^	WL condition		*t* (17)	*p*	*η*^2^	ANCOVA		
	Pre-test	Post-test				Pre-test	Post-test				*F*_(1, 33)_	*p*	*η*_*p*_^2^
DASS-21													
Depression	0.74 (0.50)	0.22 (0.33)	4.74	.000	0.54	0.45 (0.85)	0.4 (0.76)	0.73	.476	0.03	9.70	.004	0.23
Anxiety	0.57 (0.52)	0.2 (0.24)	4.05	.001	0.46	0.35 (0.70)	0.34 (0.73)	0.12	.908	0.00	7.31	.011	0.18
Stress	1.15 (0.61)	0.52 (0.43)	4.47	.000	0.51	0.64 (0.73)	0.57 (0.77)	0.72	.480	0.03	4.51	.041	0.12
Total Scale	0.82 (0.44)	0.31 (0.25)	5.65	.000	0.63	0.48 (0.74)	0.44 (0.74)	0.63	.539	0.02	10.54	.003	0.24
FSII													
Suicidal Ideation	5.4 (0.68)	5.05 (0.22)	2.33	.031	0.22	5.40 (1.06)	5.20 (0.77)	1	.334	0.07	0.57	.455	0.02

Standard deviations are presented in parenthesis. *DASS-21*, short-form version of the Depression, Anxiety, and Stress Scales; *FSII*, Frequency of Suicidal Ideation Inventory. Data about main effects for group are reported. The interaction between group and previous experience in meditation practice was not significant for all variables.

As shown in Table 5, MBI participants showed a statistically significant increase in their sleep quality (*p* < .001) and decrease in personal (*p* = .001) and work-related (*p* = .009) burnout with large effect sizes (eta squared statistic = 0.31 – 0.57). As for the control group, WL participants remained overall unchanged for sleep quality and burnout.

**Table 5:** Means, standard deviations, and paired samples *t*-test, and two-way analysis of covariance comparing sleep quality and burnout

Measure	MBI condition		*t* (19)	*p*	*η*^2^	WL condition		*t* (17)	*p*	*η*^2^	ANCOVA		
	Pre-test	Post-test				Pre-test	Post-test				*F*_(1, 33)_	*p*	*η*_*p*_^2^
PROMIS-SD													
Sleep quality	16.25 (6.09)	22.40 (4.97)	−5.04	.000	0.57	20.39 (7.41)	21.56 (8.70)	−1.241	.232	0.08	6.99	.012	0.18
CBI													
Personal burnout	2.73 (0.74)	2.1 (0.69)	4.13	.001	0.47	2.40 (1.10)	2.36 (1.17)	0.34	.736	0.01	7.99	.008	0.20
Work-related burnout	2.79 (0.84)	2.29 (0.59)	2.90	.009	0.31	2.60 (0.82)	2.60 (0.83)	−0.10	.922	0.00	7.23	.011	0.18
Client-related burnout	2.69 (0.69)	2.43 (0.74)	1.67	.112	0.13	2.11 (0.74)	2.34 (0.91)	-2.23	.039	0.23	2.93	.096	0.08

Standard deviations are presented in parentheses. *PROMIS-SD*, Patient-Reported Outcomes Measurement Information System – Sleep Disturbance; *CBI*, Copenhagen Burnout Inventory. Data about main effects for group are reported. The interaction between group and previous experience in meditation practice was not significant for all variables.

Finally, Table 6 shows a statistically significant decrease in DERS-SF Total Score (*p* = .004), as well as in the Strategies (*p* = .008), Nonacceptance (*p* = .005), and Goals (*p* = .002) subscales. The eta squared statistic indicated medium–large effect sizes for all decreases in emotion dysregulation variables (eta squared statistic = 0.08 – 0.39), except for the awareness subscale. For its part, participants from the control group did not show a significant decrease in DERS-SF scores.

**Table 6:** Means, standard deviations, and paired samples *t*-test, and two-way analysis of covariance comparing emotion dysregulation

Measure	MBI condition		*t* (19)	*p*	*η*^2^	WL condition		*t* (17)	*p*	*η*^2^	ANCOVA		
	Pre-test	Post-test				Pre-test	Post-test				*F*_(1, 33)_	*p*	*η*_*p*_^2^
DERS-SF (Total Scale)	2.23 (0.65)	1.76 (0.36)	3.25	.004	0.36	1.72 (0.76)	1.63 (0.76)	1.5	.153	0.12	1.95	.172	0.06
Strategies	2.08 (0.96)	1.48 (0.58)	2.97	.008	0.32	1.61 (0.94)	1.63 (1.05)	−0.18	.859	0.00	3.78	.060	0.10
Nonacceptance	2.15 (1.05)	1.42 (0.51)	3.15	.005	0.34	1.67 (1.01)	1.46 (0.89)	1.16	.260	0.07	2.33	.136	0.07
Impulse	1.7 (0.97)	1.43 (0.80)	1.27	.220	0.08	1.41 (0.89)	1.44 (0.86)	−0.44	.668	0.01	1.30	.262	0.04
Goals	3.32 (1.20)	2.4 (0.71)	3.49	.002	0.39	2.13 (0.98)	1.96 (1.09)	1	.331	0.06	0.22	.644	0.01
Awareness	2.13 (0.93)	2.22 (0.73)	−0.77	.449	0.03	2.04 (0.68)	1.83 (0.66)	1.38	.186	0.10	3.72	.062	0.10
Clarity	2.02 (0.73)	1.6 (0.6)	2.03	.056	0.18	1.46 (0.86)	1.42 (0.9)	0.62	.542	0.02	1.30	.263	0.04

Standard deviations are presented in parentheses. *DERS-SF*, Difficulties in Emotion Regulation Scale – Short-Form. Data about main effects for group are reported. The interaction between group and previous experience in meditation practice was not significant for all variables.

Regarding comparisons between groups, there was no significant interaction effect between group and previous experience in meditation practice for any variable. These results suggest that people who had some previous experience did not respond differently to the MBI/WL than those who had not. Two-way ANCOVA results are reported throughout Tables 3, 4, 5, and 6 along with means, standard deviations, and paired samples *t*-test.

After adjusting for pre-test scores, there was a statistically significant difference between the two groups on post-intervention scores on the FFMQ-SF Observe facet (*p* = .006; partial eta squared = 0.21), DASS-21 Depression subscale (*p* = .004; partial eta squared = 0.23), DASS-21 General Distress Index (*p* = .003; partial eta squared = 0.24), and CBI Personal Burnout subscale (*p* = .008; partial eta squared = 0.20).

The differences between groups on the FFMQ-SF Total Score, SCS-SF Total Score, SCS-SF Positive subscale, DASS-21 Anxiety, DASS-21 Stress, Sleep Quality, CBI Work-related burnout, DERS-SF Strategies, and DERS-SF Awareness showed large effect sizes, though there were no statistically significant differences (*p* > .01). Thus, hypothesis 2 was partially supported.

Specifically on frequency of suicide ideation, results showed a statistically non-significant decrease in frequency of suicide ideation (*p* = .031) with large effect sizes (eta squared statistic = 0.22) in the MBI group, while the control group remained unchanged (see Table 4). Furthermore, the differences between groups in frequency of suicide ideation were not statistically significant (*p* = .455; partial eta squared = 0.02). Thus, hypothesis 3 was not supported.

Linear mixed effects modelling of emotions showed a main effect of Week on anger (*p* = .005), disgust (*p* = .009), anxiety (*p* < .001), sadness (*p* < .001), and desire (*p* < .001), which significantly decreased over the intervention period. There was no statistically significant difference in fear (*p* = .053), relaxation (*p* = .783), and happiness (*p* = .176) for the 8 weeks. Thus, hypothesis 4 was partially supported. See Table 7 for descriptive statistics and linear mixed model results.

**Table 7:** Means of state emotions, standard deviations, and the linear mixed model results

Measure	Week 1	Week 2	Week 3	Week 4	Week 5	Week 6	Week 7	Week 8	*df*	*F*	*p*
Anger	2.13 (0.72)	1.84 (0.68)	1.79 (0.76)	1.63 (0.62)	1.54 (0.75)	1.65 (0.73)	1.56 (0.66)	1.63 (0.73)	7,127.12	3.08	.005
Disgust	1.84 (0.72)	1.64 (0.80)	1.64 (0.83)	1.43 (0.52)	1.38 (0.50)	1.35 (0.37)	1.09 (0.44)	1.31 (0.33)	7,127.39	2.82	.009
Fear	1.57 (0.71)	1.28 (0.46)	1.28 (0.41)	1.25 (0.37)	1.37 (0.59)	1.24 (0.29)	1.23 (0.34)	1.25 (0.38)	7,127.35	2.06	.053
Sadness	2.68 (1.30)	2.36 (1.05)	2.10 (0.82)	2.06 (0.91)	1.95 (0.90)	1.80 (0.84)	1.66 (0.66)	2.16 (1.10)	7,126.99	4.62	.001
Happiness	4.71 (0.96)	4.95 (0.99)	4.93 (1.06)	4.71 (1.24)	4.79 (1.21)	5.07 (1.14)	4.88 (1.30)	4.69 (1.30)	7,127.05	1.49	.176
Anxiety	3.20 (0.84)	2.60 (0.80)	2.50 (0.75)	2.16 (0.69)	2.36 (0.86)	2.09 (0.77)	2.03 (0.84)	2.24 (0.82)	7,127.16	9.58	.001
Desire	3.66 (0.87)	3.31 (0.80)	2.78 (0.78)	2.89 (0.76)	2.87 (0.96)	2.64 (0.81)	2.69 (1.17)	2.84 (1.19)	7,127.01	8.74	.001
Relaxation	4.73 (0.79)	4.81 (0.95)	4.78 (1.10)	4.74 (1.18)	4.93 (1.21)	4.98 (1.08)	5.03 (1.24)	4.84 (1.17)	7,127.08	0.57	.783

Standard deviations are presented in parentheses

**Figure 2:**
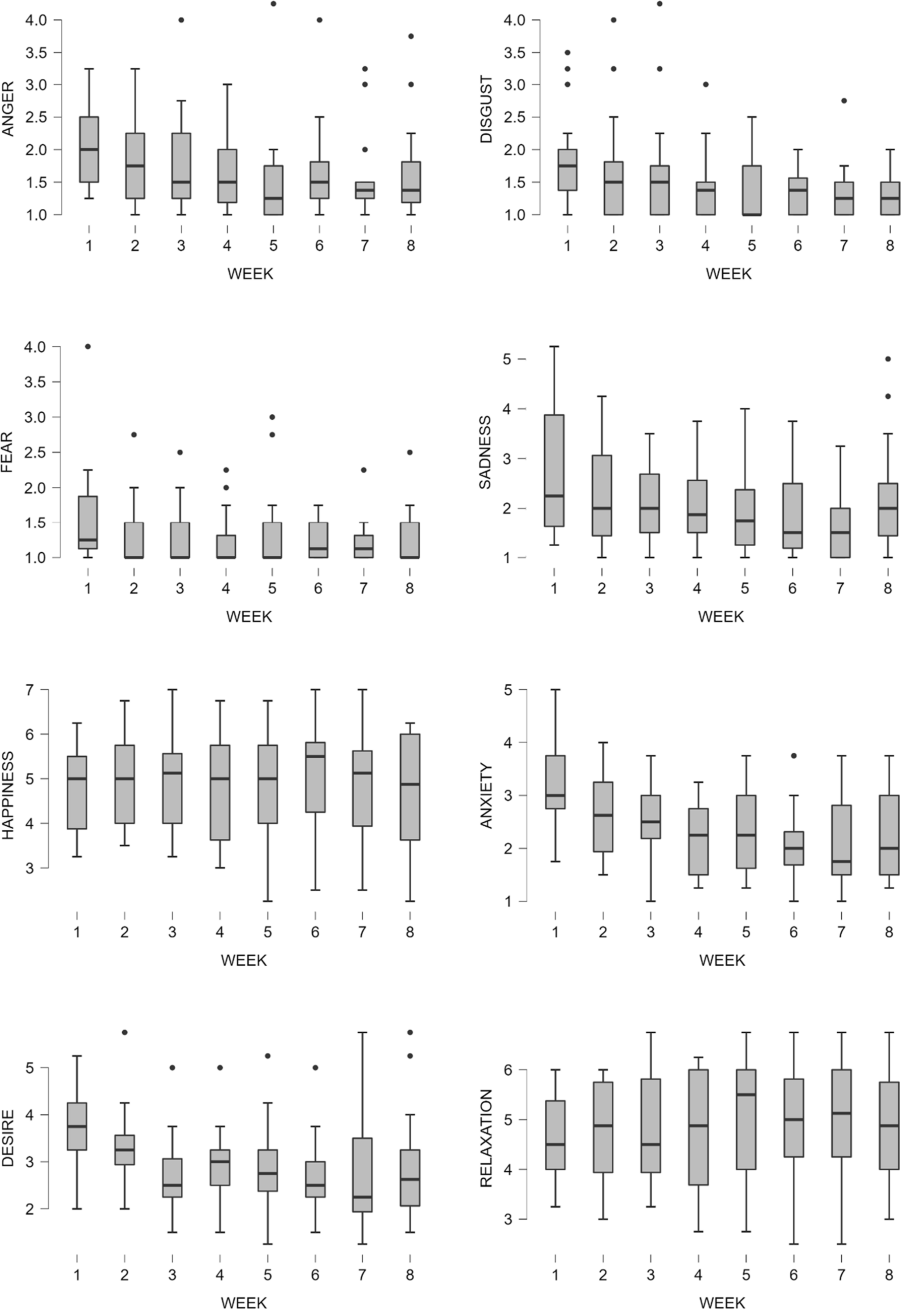
Self-report emotion state of MBI participants during the 8 weeks

Figure 2 shows the self-report emotion state of MBI participants during the 8 weeks. Post hoc comparisons using Bonferroni adjustment to the alpha level indicated statistically significant decreases on anger in weeks 4, 5, 6, and 8, disgust in week 8, anxiety in weeks 2 – 8, sadness in weeks 3 –7, and desire in weeks 3 –8 (see Table 8 for more details).

**Table 8:** Post hoc comparisons of state emotions between week 1 and weeks 2–8.

Measure	W2 –W1		W3 –W1		W4 –W1		W5–W1		W6–W1		W7 – W1		W8 –W1	
	*p*	*η*^2^	*p*	*η*^2^	*p*	*η*^2^	*p*	*η*^2^	*p*	*η*^2^	*p*	*η*^2^	*p*	*η*^2^
Anger	1	0.04	.705	0.05	.027	0.10	. 004	0.14	.047	0.09	.118	0.13	.027	0.10
Disgust	1	0.02	1	0.02	.239	0.07	.133	0.09	.057	0.10	.185	0.20	.026	0.11
Fear	.297	0.04	.297	0.04	.157	0.04	1	0.05	.112	0.02	.073	0.05	.157	0.04
Sadness	1	0.01	.042	0.04	.022	0.05	.007	0.07	.000	0.10	.001	0.12	.114	0.03
Happiness	1	0.01	1	0.01	1	0.00	1	0.00	1	0.03	1	0.01	1	0.00
Anxiety	.010	0.11	.001	0.14	.000	0.26	.000	0.19	.000	0.29	.000	0.31	.000	0.23
Desire	.317	0.04	.000	0.19	.000	0.15	.000	0.16	.000	0.24	.000	0.22	.000	0.17
Relaxation	1	0.00	1	0.00	1	0.00	1	0.01	1	0.02	1	0.03	1	0.00

Eta squared (*η*^2^) indicates the proportion of variance explained by the independent variable and values range from 0 to 1. Cutoff values are 0.01, 0.06, and 0.14 for small, medium, and large effect sizes, respectively.

## Discussion

There is growing evidence that supports the preliminary effectiveness of MBIs delivered to police officers, a group exposed to frequent risky situations and stressful events. Studies in this field, however, are still scarce. The purpose of this exploratory non-randomized study was to determine the acceptance by police officers and preliminary effectiveness of a MBI, which was created to judiciously increase attentional deployment, body awareness, emotion regulation strategies, and a detached perspective of the self (Cebolla et al., 2018).

Participants showed high acceptance and attendance to the intervention sessions (supporting Hypothesis 1). Related to this, significant factors that might positively impact the functioning of MBIs have been met in this study, such as a within-group sense of safety, cohesion, and support and alliance (Hutchinson et al., 2021). In this regard, the present psychological intervention for police officers was designed and implemented by a police officer (along with a psychologist). We suppose that the therapeutic alliance was strengthened by the fact that one of the instructors was a member of the National Police Corps. This relational bond between mindfulness instructors and clients has been positively associated with mindfulness, negative affect, and emotion regulation post-training outcomes, among other variables (Goldberg et al., 2013; Jazaieri et al., 2018). However, Christopher et al., (2016, 2018), Grupe et al. (2021), and Márquez et al. (2021) reported similar acceptance, attendance, and dropouts than us, so this might not be a determinant factor, at least considering quantitative data. In addition, frequency of home practice was acceptable, with an average of 23 min per day, 5 days per week. This is in line with the frequency of practice in the context of Mindfulness-based Stress Reduction program (Kabat-Zinn, 2009) and Mindfulness-Based Cognitive Therapy (Segal et al., 2002), in which participants practice 29 min per day, 6 days per week (Parsons et al., 2017).

Moreover, it was found that the police officers who underwent the MBI significantly increased mindfulness, self-compassion, and sleep quality, as well as decreased their difficulties in emotion regulation, depression, anxiety, stress, and burnout. These results are in line with those obtained by Christopher et al. (2016), Grupe et al. (2021), and Márquez et al. (2021). More specifically, the participants decreased their difficulties in emotion regulation assessed with the DERS, which is consistent with the results of Christopher et al. (2016). Participants especially increased the belief about their capability to regulate emotions effectively (Strategies), their acceptance instead of denial of negative emotions (Nonacceptance), and their engagement in goal-directed behaviors while experiencing negative emotions (Goals). Moreover, the study of Márquez et al. (2021) was the only one of them to include a compassion measure, though contrary to the present results no significant differences were found. However, these results partially supported Hypothesis 2, since there were no significant differences for the rest of the outcomes.

Significant between-group differences were also found in the way of attending to internal and external experiences (Observing mindfulness facet), depression symptoms, general distress, and the degree of physical and psychological exhaustion (personal burnout) of MBI participants versus the control group (partially supporting Hypothesis 2). Similarly, Christopher et al. (2018) included a no intervention control group in their randomized trial too. These authors showed that participants decreased their burnout, organizational stress, aggression, and alcohol use, and increased both non-reactivity to internal experience (Nonreactivity mindfulness facet) and psychological flexibility. Furthermore, the present results are in line with previous studies, which have shown the effectiveness of mindfulness training on improving emotional exhaustion and job satisfaction (burnout dimensions) in emotionally demanding jobs (e.g., Hülsheger et al., 2013; Luken & Sammons, 2016).

Regarding frequency of suicide ideation, there were no statistically significant within-/between-groups differences (rejecting Hypothesis 3). However, police officers’ baseline levels were low for both conditions, suggesting a floor effect. Also, the magnitude of the differences on the means of the MBI group was large. Taking into account the relatively small sample and associated low statistical power, we still think this hypothesis is worthy of further consideration in future studies. On the one hand, first responders are a population with a high tendency to experience suicide ideation and elevated risk of suicide (Stanley et al., 2016; Violanti & Steege, 2021). Several studies support the determinant implication of emotion (dys)regulation in the development of suicidal ideation. For instance, Law et al. (2015) reported that individuals who experience high emotion dysregulation are more likely to ideate and commit suicide. In this line, emotion dysregulation might be a risk factor for suicide given how it predicts engagement in non-suicidal self-injury behaviors (Heffer & Willoughby, 2018). On the other hand, the theoretical rationale of MBIs for suicidal ideation and behavior is based on the evidence of their effectiveness in improving attentional control, awareness, problem solving, and stress response, which are common dysregulated areas in individuals who have attempted suicide (Chesin et al., 2016; Forkmann et al., 2016).

Finally, participants of the MBI group experienced a week by week decrease of anger, disgust, anxiety, sadness, and desire, meanwhile fear, relaxation, and happiness remained overall unchanged (partially supporting Hypothesis 4). These results are consistent with the evidence regarding the effectiveness of MBIs on reducing negative affectivity (Khoury et al., 2015; Schumer et al., 2018). In addition, these changes are in line with a recently proposed biopsychosocial model of stress and health in policing that highlights the relevance of appraisal and emotional regulation-based strategies to cope well with stress (Gutschmidt & Vera, 2021). Furthermore, Christopher et al. (2016) reported a significant pre-/post-test reduction of police officer’s anger levels too. In this regard, Bergman et al. (2016) found that increases in mindfulness facets predict reductions of anger and stress in police officers, which might be due to the mediator role of mindfulness between rumination and anger (Borders et al., 2010).

In light of the above, MBI is potentially an effective way to prevent stress resulting from police officers’ daily work and to increase their quality of life. Only few studies in recent years have examined potential effects of MBIs on the mental health of police officers (e.g., Hoeve et al., 2021; Trombka et al., 2021), and the present one replicated some of their findings. Not only that, but the present study increased the generalizability of their results by conducting an MBI in a country whose police corpses are still unaware of the potential benefits of this kind of interventions (Márquez et al., 2021). Moreover, the frequency of suicide ideation was included among the outcomes as a small step towards the in-depth study of the potential contribution of MBIs on police suicides, which seems a general concern (e.g., Violanti & Steege, 2021). Furthermore, the weekly assessment showed valuable data about the emotion state evolution of police officers involved in the intervention. This procedure was not carried out in previous studies in the field and the data collected by this way enriched the information obtained from the pre-/post-assessments. Finally, counting on a police officer as a member of the research team may have facilitated the recruitment, though quantitative analyses could not report it. In that regard, the psycho-educational context (versus clinical or spiritual) in which the present and most of the studies in mindfulness training for police officers were developed requires including mindfulness instructors who are also police experts (Cebolla & Campos, 2016).

### Limitations and Future Research

This study is not exempt of limitations. Mainly, the sample size was small, thus limiting the statistical power of the work and generalization of inferences. Also, we did not randomly assign participants to the groups because of the pilot nature of this study, so the causality of the results is not guaranteed. Furthermore, we did not expect that so many people were interested in participating in the study. In this line, due to a potential self-selection of the sample, results could be positively biased. Moreover, COVID safety measures during the intervention might have influenced the group dynamics, thus biasing the present findings. For example, the quality of in-session meditations might have been affected by them (e.g., meditating with a face mask). Furthermore, state emotions were not measured in the control group so only within-group comparisons were made. In addition, the groups were evaluated using different methods (in-person versus online assessment), which might have implied a method bias related to measurement context effects (Podsakoff et al., 2003). Finally, the study protocol was not publicly available nor previously registered on any platform.

Future research is needed to test the efficacy of this MBI by developing a randomized, controlled design. For that purpose, the present study brings the opportunity to properly calculate the sample size. In addition, the use of biological variables previously reported in MBI research, such as salivary immunoglobulin A (Bellosta-Batalla et al., 2018), would strengthen and support data from self-reported measures. In this line, qualitative information would also be useful to complement the conclusions that can be drawn from quantitative data. Finally, follow-up assessments are required to investigate long-term effectiveness of the intervention.

## Data Availability

The data that support the findings of this study are available on request from the corresponding author. The data are not publicly available due to a privacy issue.
